# A novel BACE inhibitor NB-360 shows a superior pharmacological profile and robust reduction of amyloid-β and neuroinflammation in APP transgenic mice

**DOI:** 10.1186/s13024-015-0033-8

**Published:** 2015-09-03

**Authors:** Ulf Neumann, Heinrich Rueeger, Rainer Machauer, Siem Jacob Veenstra, Rainer M. Lueoend, Marina Tintelnot-Blomley, Grit Laue, Karen Beltz, Barbara Vogg, Peter Schmid, Wilfried Frieauff, Derya R. Shimshek, Matthias Staufenbiel, Laura H. Jacobson

**Affiliations:** Neuroscience, Novartis Institutes for BioMedical Research (NIBR), Basel, Switzerland; Global Discover Chemistry, NIBR, Basel, Switzerland; Metabolism and Pharmacokinetics, NIBR, Basel, Switzerland; DMPK, NIBR, Basel, Switzerland; Swiss Tropical and Public Health Institute, Basel, Switzerland; Preclinical Safety, NIBR, Basel, Switzerland; Hertie Institute for Clinical Brain Research, Tübingen, Germany; The Florey Institute of Neuroscience and Mental Health, The University of Melbourne, Parkville Melbourne, Australia

**Keywords:** Alzheimer’s disease, BACE-1, Inhibitor, Amyloid-β, Neuroinflammation

## Abstract

**Background:**

Alzheimer’s disease (AD) is the most common form of dementia, the number of affected individuals is rising, with significant impacts for healthcare systems. Current symptomatic treatments delay, but do not halt, disease progression. Genetic evidence points to aggregation and deposition of amyloid-β (Aβ) in the brain being causal for the neurodegeneration and dementia typical of AD. Approaches to target Aβ via inhibition of γ-secretase or passive antibody therapy have not yet resulted in substantial clinical benefits. Inhibition of BACE1 (β-secretase) has proven a challenging concept, but recent BACE1inhibitors can enter the brain sufficiently well to lower Aβ. However, failures with the first clinical BACE1 inhibitors have highlighted the need to generate compounds with appropriate efficacy and safety profiles, since long treatment periods are expected to be necessary in humans.

**Results:**

Treatment with **NB-360**, a potent and brain penetrable BACE-1 inhibitor can completely block the progression of Aβ deposition in the brains of APP transgenic mice, a model for amyloid pathology. We furthermore show that almost complete reduction of Aβ was achieved also in rats and in dogs, suggesting that these findings are translational across species and can be extrapolated to humans. Amyloid pathology may be an initial step in a complex pathological cascade; therefore we investigated the effect of BACE-1 inhibition on neuroinflammation, a prominent downstream feature of the disease. **NB-360** stopped accumulation of activated inflammatory cells in the brains of APP transgenic mice. Upon chronic treatment of APP transgenic mice, patches of grey hairs appeared.

**Conclusions:**

In a rapidly developing field, the data on **NB-360** broaden the chemical space and expand knowledge on the properties that are needed to make a BACE-1 inhibitor potent and safe enough for long-term use in patients. Due to its excellent brain penetration, reasonable oral doses of **NB-360** were sufficient to completely block amyloid-β deposition in an APP transgenic mouse model. Data across species suggest similar treatment effects can possibly be achieved in humans. The reduced neuroinflammation upon amyloid reduction by **NB-360** treatment supports the notion that targeting amyloid-β pathology can have beneficial downstream effects on the progression of Alzheimer’s disease.

## Background

Alzheimer’s disease (AD) is the most common form of senile dementia, with increasing incidence as a function of age. It poses a significant challenge to the healthcare systems in both industrial and developing countries. Major efforts to find disease-modifying therapies have focused on the key pathological alterations in AD brains, aggregation of β-amyloid (Aβ) and tau protein aggregation. Aβ peptides are being constantly generated from the precursor APP in the normal brain but their concentrations remain low due to the presence of effective clearance mechanisms. During disease development, insoluble forms accumulate in cortical and subcortical brain areas. Substantial evidence supports the concept that neurotoxicity of aggregated Aβ isoforms and/or Aβ-induced neuroinflammation are triggering neurodegeneration in AD [1]. Various therapeutic approaches have been developed to prevent generation or aggregation of Aβ in the brain, or to remove insoluble Aβ. The clinically most advanced approach is Aβ immunotherapy, with a number of phase 3 trials completed, however, data to date have showed no or only marginal disease modification [2, 3]. Similar disappointing results have come from randomized clinical trials of anti-inflammatory drugs, despite promising data from epidemiological findings [4]. Negative results of Aβ immunotherapy studies in particular have led to the concept that Aβ targeting therapeutics may be most efficacious at the pre-clinical stages of the disease. This is supported by recent findings from longitudinal studies indicating that Aβ accumulation may be present decades before disease manifestation [5]. Together this supports prevention of Aβ formation as a strategy for investigation.

Generation of Aβ may be blocked by inhibiting BACE-1 (β-site APP Converting Enzyme 1) or γ-secretase [6, 7]. Inhibition of γ-secretase has faced serious safety issues, while drug-like BACE inhibitors were not found within the chemical space of first generation hydroxyethylene and ethanolamine scaffolds. The discovery of the cyclic amidine motif represented a major step forward in BACE inhibitor development (for a recent overview: [8]), as these rigid and compact structures allowed designing molecules with improved targeting of BACE-1 in the brain. Several clinical candidates have emerged from this second wave of BACE-1 inhibitors, and have been investigated in preclinical toxicological studies and in early clinical trials. Compounds MK-8931, AZ-3293, E-2609, and JNJ-54861911 moved into AD trials, but their characteristics remain largely unpublished. Another set of compounds failed to reach long term clinical trials mainly due to safety issues, including cardio safety [9], liver marker changes [10], and retinal toxicity [11, 12]. Incorporating the learnings from these failures into the design of new BACE-1 inhibitors therefore offers the chance to develop a 3^rd^ generation of compounds with an improved safety profile, allowing long term treatment of AD patients.

In order to address these problems, our research has focused on three aspects: (1) an improved metabolism pattern by introducing a novel scaffold. (2) Excellent brain penetration, as reduction of the peripheral exposure (the “body burden”) is expected to improve the overall safety profile. (3) Very good selectivity over cathepsins D and E.

**Fig. 1:**
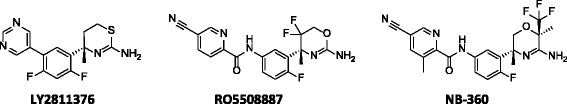
Structure of LY2811376, RO5508887, and NB-360

We discovered a novel structural class of BACE-1 inhibitors, characterized by the amino-1, 4-oxazine core and here describe compound **NB-360** (Fig. 1). Our data on efficacy *in vitro* and *in vivo*, in various preclinical models, as well as on pharmacokinetics and brain penetration in animal models show favorable potency and excellent distribution properties. When investigated in acute and chronic studies in rodent and non-rodent models, **NB-360** substantially reduced soluble and deposited forms of amyloid-β and showed reduction of neuroinflammation markers.

## Results

### NB-360 potency, selectivity, and brain penetration

**NB-360** was tested in a series of enzyme inhibition assays with relevant aspartyl proteases. Potency against the mouse BACE-1 was equal to the human enzyme (IC_50_ for both: 5 nM), and equipotent inhibition of BACE-2 was observed (IC_50_: 6 nM). **NB-360** did not inhibit pepsin or the endosomal/lysosomal cathepsins D and E (IC_50_ > 250 μM). **NB-360** was a potent inhibitor of Aβ40 release from human APP-overexpressing CHO cells, with an IC_50_ of 3 nM in wtAPP CHO cells and 33 nM in SweAPP CHO cells. The release of Aβ42 (IC_50_: 3 nM) and sAPPβ (IC_50_: 4 nM) in wild-type APP CHO cells was inhibited with similar potency as the release of Aβ40.

We next investigated the ability of **NB-360** to cross the blood-brain-barrier and reach its target in the brain. *In vitro* assays showed moderate to high lipophilicity and high passive permeation in an artificial membrane model (PAMPA model, [13]). We used a cellular transport model (MDR1-MDCK cells) to test for a potential recognition of **NB-360** by one of the major efflux transporters in the blood brain barrier, p-glycoprotein [14]. We found high apical-to-basolateral permeation; the p-glycoprotein mediated transport in the basolateral-to-apical direction was only slightly higher. This indicated that p-glycoprotein mediated efflux was unlikely to play a major role for tissue distribution of **NB-360**. Non-specific binding to plasma proteins and brain homogenate was high (Table 1).

**Table 1:** Physico-chemical and transport properties of NB-360

Molecular weight	449.5
logP (octanol/water)	3.7
Dissociation constant (pKa)	7.1
Passive membrane permeability (logPe pH 6.8)	-3.6 cm s^-1^
MDR1- MDCK flux apical-basolateral (A-B)	14.1• 10^-6^ cm s^-1^
MDR1- MDCK flux basolateral-apical (B-A)	26.3• 10^-6^ cm s^-1^
MDR1- MDCK efflux ratio (B-A/A-B)	1.9
Plasma protein binding (rat)	93.8 %
Plasma protein binding (dog)	>99 %
Non-specific brain homogenate binding (rat)	97.7 %

*In vivo* blood pharmacokinetics and brain penetration was investigated in the rat. Animals were orally dosed with 30 μmol/kg (13 mg/kg) **NB-360,** suspended in methylcellulose 0.5 % w/v in water/0.1 % Tween 80 v/v) and killed at 5 time points between 1 and 24 h. Blood and brain were collected and analyzed for **NB-360** concentrations. Tmax in blood was 1 h, and the associated Cmax together with the AUC-values for total and unbound compound in the blood and in the brain compartment are shown in Table 2. While the total concentration of **NB-360** in the brain was approximately 2-fold higher than in the blood, after correction for nonspecific binding the unbound concentrations were very similar. This indicated that the **NB-360** pool in the peripheral and in the central compartment was in equilibrium, and that efflux at the blood-brain-barrier did not play a significant role in compound distribution.

**Table 2:** NB-360 concentrations in rat blood and brain, after a 30 μmol/kg oral dose

	Blood	Brain
Cmax (1 h, μM)	1.62	4.01
AUC 0-24 h (μM•h)	10.2	23.6
unbound AUC 0-24 h (μM•h)	0.63	0.54

### NB-360 pharmacokinetics

The pharmacokinetic parameters after intravenous and oral application of **NB-360** were investigated in mice, rats and beagle dogs and are summarized in Table 3. Clearance was low in the mouse and higher in the rat. The clearance from dog blood was very slow and the volume of distribution was remarkably low, in comparison to the other species. Both parameters may be linked to the very high plasma protein binding in dogs (Table 1). Oral bioavailability was moderate to high in all species; these data supported the use of oral dosing in further pharmacological experiments.

**Table 3:** Pharmacokinetic parameters of NB-360 after oral and intravenous application in mouse, rat and beagle dog

Parameter	Mouse	Rat	Dog
Dose i.v. (μmol/kg)	2	2	2
Dose p.o. (μmol/kg)	6	6	2
Clearance after i.v. application (ml/min/kg)	16	52	0.4
Terminal half-life after i.v. application (hours)	3.6	2.7	36.2
Vol. of Distribution (l/kg)	4.6	10.2	0.9
Oral Bioavailability (%)	37	92	62

### Pharmacokinetics/pharmacodynamics studies of NB-360 in rats

**Fig. 2:**
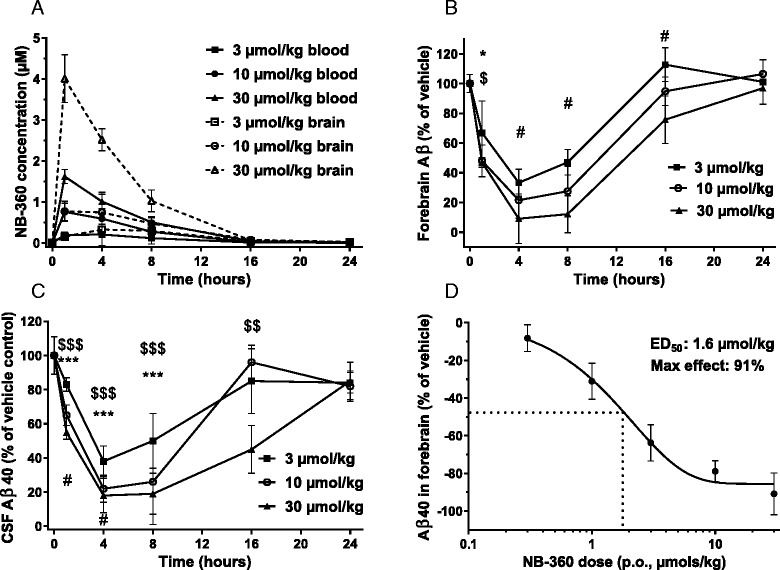
Pharmacokinetics and pharmacodynamics of NB-360 in rats after oral dosing. The significance of differences in effect sizes (analyzed as % of vehicle) produced by the doses within each timepoint was tested. **a**-**c**: 3 μmol/kg (squares), 10 μmol/kg (circles), and 30 μmol/kg (triangles). Mean values ± SD are shown, n = 5. **a** NB-360 exposure in blood and brain. **b** Aβ40 in forebrain, *p < 0.05 3 vs 10 μmol/kg, $ p < 0.05 3 vs 30 μmol/kg, # p < 0.01 3 vs 30 μmol/kg **c**: Aβ40 in CSF, ***p < 0.001 3 vs 10 μmol/kg, $ p < 0.05, $$ p < 0.01, $$$ p < 0.001 3 vs 30 μmol/kg, # p < 0.05 and ### p < 0.001 10 vs 30 μmol/kg, **d**: Dose-response in brain based on 4 h data

**NB-360** was orally dosed to rats at 3, 10, and 30 μmol/kg, and compound concentrations were measured at different time points over 24 h in blood and in forebrain (Fig. 2a). **NB-360** was rapidly absorbed with a Tmax around 1 h, and showed a parallel decline in blood and brain over time. Aβ40 was determined in the brain homogenate and in CSF (Fig. 2b and 2c), and a dose- and time-dependent reduction was observed in both tissues. Maximum activity was between 4 and 8 h, and Aβ40 concentrations returned to baseline after 24 h. Significant (>50 %) Aβ40 lowering was observed even at the lowest dose of 3 μmol/kg, and the effect saturated at higher doses. At the 30 μmol/kg dose, 90.9 % inhibition was reached at 4 h, corresponding to 0.06 pmol Aβ40/mg brain tissues. Although we did not perform a formal determination of the quantification limit of Aβ40 in rat brain, previous experience indicated that this was the practical limit of quantification under our extraction and analysis conditions. Since **NB-360** showed such a profound inhibition even at the 3 μmol/kg p.o. dose, in a second study we extended the dose range to 0.3 and 1 μmol/kg and calculated an efficacious dose (ED_50_) of 1.6 ± 0.25 μmol/kg for inhibition of forebrain Aβ40 in the rat at 4 h (Fig. 2d). Overall, **NB-360** was highly potent in the rat, and compound exposure correlated well to effect.

### Effects of NB-360 in dogs

**Fig. 3:**
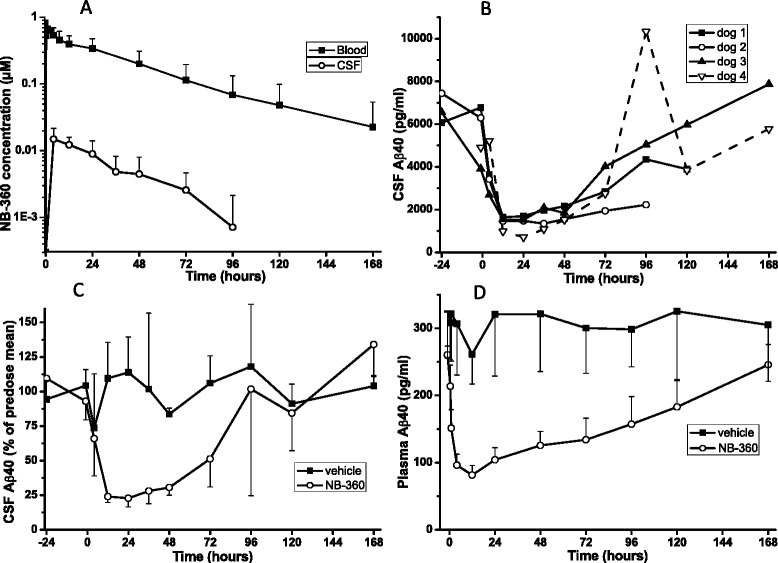
Pharmacokinetics and pharmacodynamics of NB-360 in Beagle dogs. **a** Exposure of NB-360 in blood and CSF of cannulated dogs (mean ± SD, n = 4) after 0.5 mg/kg p.o. dosing, **b** concentrations of CSF Aβ40 for the individual dogs, late CSF samples were missing for dogs 1 and 2, **c** CSF Aβ40 in vehicle and treatment group, expressed as percent of the mean of the -24 h and the -1 h predose values (mean ± SD), **d** plasma Aβ40 in vehicle and treatment groups

We selected dogs to evaluate the **NB-360** pharmacology in a non-rodent animal model because they are commonly used for toxicological investigations, and are discussed as models for human AD [15]. Furthermore dog and human Aβ show sequence identity. Our pharmacokinetic studies in dogs (Table 3) showed good oral bioavailability and much longer half-life, compared to rodents. Based on pharmacokinetic data, we selected a dose of 0.5 mg/kg (1.1 μmol/kg) p.o. for a pharmacokinetic/pharmacodynamic study of **NB-360** in young Beagle dogs, carrying an implanted ventricular port for repeated CSF sampling. Blood and CSF were sampled before treatment (at -24 and at -1 h) and at various time points after oral dosing, up to 168 h. The pharmacokinetic profiles of **NB-360** in blood and in CSF were very similar (Fig. 3a). The peak concentration of 0.7 μM was reached in blood quickly after dosing. At the 168 h time point, **NB-360** concentrations were 0.023 ± 0.031 μM in blood, and below detection limit in CSF. The average CSF/blood ratio was 0.023 ± 0.014 (mean ± SD, calculated from n = 20 CSF/blood data pairs). Without radiolabeled **NB-360** being available, we were unable to accurately determine the plasma protein binding in dog plasma, Therefore, a precise calculation of the CSF/free blood ratio could not be done.

The mean (± SD) level of Aβ40 in the CSF prior to dosing was 5991 ± 1199 pg/ml (2 sampling times -24 h and -1 h, n = 7), and the predose plasma Aβ40 level was 261 ± 13 pg/ml (mean ± SD, n = 4, -1 h). A single *per os* administration of **NB-360** treatment at the dose of 0.5 mg/kg caused a rapid and long-lasting drop in Aβ concentration in dog CSF and plasma. All dogs responded strongly to the treatment, CSF Aβ40 was reduced by approximately 80 % from 12 to 48 h post dose and slowly returned to baseline (Fig. 3b). Baseline was reached at about 120 h after the dose. Vehicle-treated dogs did not show a significant change of CSF Aβ40 values (Fig. 3c). Plasma Aβ40 levels responded very quickly (-42 % after 1 h) to **NB-360** treatment, the effect reached -70 % after 12 h and very slowly returned towards baseline, which was not fully reached even 168 h after the dose (Fig. 3d). Using PK/PD modelling, we calculated an *in vivo* IC_50_ of 59 ± 13 nM of **NB-360** in blood for the reduction of Aβ40 in CSF. Furthermore, the available data were used for a calculation of the Aβ40 clearance rate from dog CSF. We obtained a clearance rate constant k = 0.26 ± 0.07 h^-1^, corresponding to a half-life of 3.7 h.

### Chronic NB-360 treatment in APP51/16 transgenic mice

To find a dosing regimen for chronic treatment, **NB-360** was first orally dosed to young (3-5 months) pre-plaque APP51/16 mice which harbor the human APP751 gene under the control of the murine Thy1 promotor. This strain of transgenic mice was selected since it resembles the situation in sporadic human AD, where no APP mutation is present. Four hours after a 30 μmol/kg single dose, we observed 79.6 % (n = 5, p = 0.0006) lowering of TX-100 soluble brain Aβ40, in good agreement with the results obtained in rats. Next, we dosed the compound p.o. once daily for 4 days at 2 doses to pre-plaque APP51/16 mice (age: 8 months) in order to achieve steady state exposure, and measured the Aβ40 reduction 24 h after the last dose. When dosing 30 μmol/kg/d, we observed 15.1 % (n = 5, p = 0.02 *vs* vehicle) reduction of forebrain Aβ. At the 100 μmol/kg/d dose, Aβ40 reduction after 24 h was 52.1 % (p = 0.004). In order to achieve a >50 % reduction of brain Aβ over a 24 h period in mice we selected this dose for a chronic study.

Female APP51/16 mice at 14.5 month of age (within the exponential phase of plaque growth) were treated once daily with 100 μmol/kg p.o. of **NB-360** for 6 weeks. Animals in the baseline group were killed at the age of 14.5 months, before start of dosing. Two out of 17 mice in the treatment group died during treatment. This level of loss is within normal ranges for this strain and age of mice. Mice of the vehicle and treatment groups were killed at 16 months of age, 4 and 24 h after the last dose and blood, CSF, and brain were collected. During the study, we noted the occurrence of patches of grey hair in the mice of the treatment group. They first appeared after 2-3 weeks of treatment and affected all mice within the treatment group but to a variable degree.

### NB-360 exposure and effect on APP metabolites in CSF and plasma

**Fig. 4:**
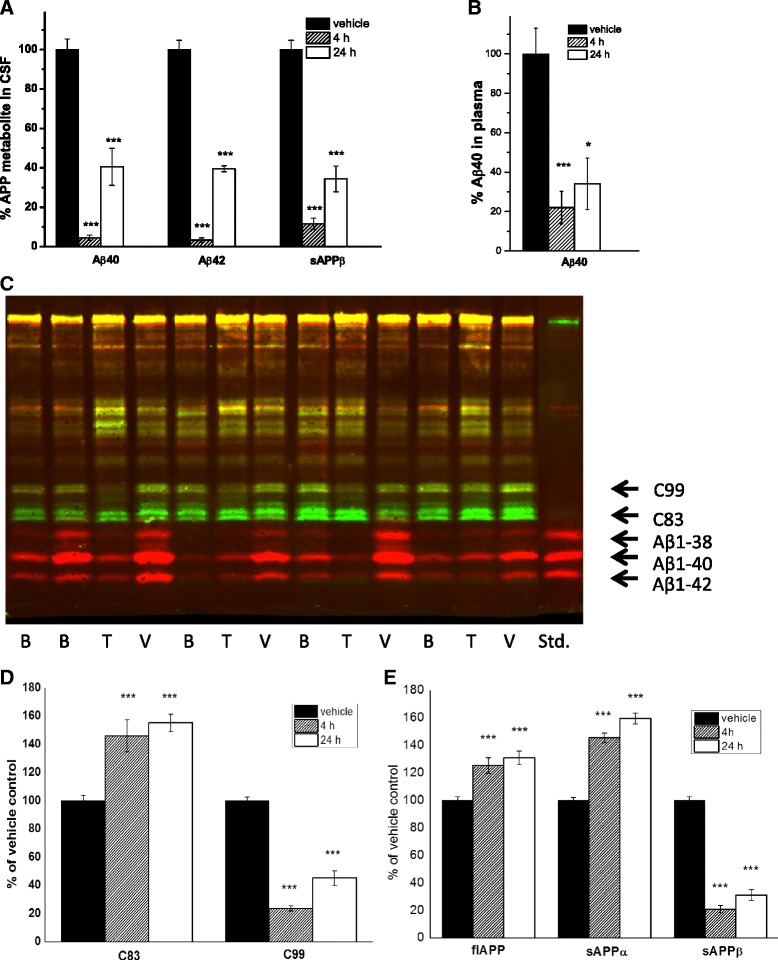
Effect of NB-360 on APP metabolites in APP51/16 mice, measured in Triton X100 extract 4 and 24 h after the last dose of a 6 week study (100 μmol/kg/d p.o.). **a** Aβ40, Aβ42, and sAPPβ in CSF, **b** Aβ40 in plasma, **c** Example of forebrain samples on Western blot stained with C-terminal APP antibody (green) and N-terminal Aβ antibody (red). B: baseline, 14.5 mo, V: vehicle 16 mo, T: treatment with NB-360, **d** quantification of Western blot data for C83 and C99. **e** MSD-immunoassay results for full-length APP, sAPPα, and sAPPβ in TX-100 forebrain extract. Data are means ± SEM, n = 8-17, all comparisons done against vehicle group

**NB-360** exposure in APP51/16 mouse blood was 3.2 μM at 4 h after the last dose, and declined to 0.13 μM over the next 20 h. Concentrations of soluble APP metabolites in the CSF were determined at 4 and 24 h. The effect on the BACE-1 dependent APP metabolites correlated with exposure, with Aβ40 and Aβ42 showing > 90 % reduction 4 h after the dose, and about 60 % at 24 h. Soluble APPβ (sAPPβ) had a slightly different profile, with less pronounced reduction at 4 h, but stronger effect towards 24 h (Fig. 4a). As an overall measure of daily efficacy within a repeated dosing study, we calculated an approximate area under the effect curve for the 24 h period after the last dose (AUEC_24h_ from 0, 4, 24 h time points). CSF Aβ40, Aβ42, and sAPPβ showed almost identical efficacy of approximately 75 % reduction. Plasma Aβ40 showed a lower efficacy at the 4 h time point, with an overall AUEC_24h_ of 70 % reduction (Fig. 4b).

### Effect of chronic NB-360 treatment on APP metabolites in Triton TX-100 extracted forebrain

The effects of **NB-360** on the C-and N-terminal APP fragments were quantified in Triton TX-100 forebrain extracts. C99 and sAPPβ are the membrane-bound and soluble metabolites, respectively, originating from BACE-1 cleavage before amino acid 1 of the Aβ sequence. Both showed about 80 % reduction, 4 h after the last dose, and the effect returned to 70 % (sAPPβ) and 57 % (C99) reduction at 24 h (Fig. 4c). Unprocessed, full-length APP as well as the α-secretase pathway products C83 and sAPPα moderately increased as a consequence of **NB-360** treatment. Their concentrations did not change considerably between 4 and 24 h, and an average increase of 30 % (full-length APP), and 50 % (sAPPα and C83) above vehicle control was observed (Fig. 4d).

### Effect of chronic NB-360 treatment on Aβ peptides in formic acid extracted forebrain and amyloid plaques in cerebral cortex

At treatment start, the baseline group of APP51/16 mice showed moderate amounts of formic acid (FA)-soluble Aβ38, 40 and 42. Mice dosed with vehicle had more than double the amount of FA-soluble Aβ within the 6 weeks period. Mice treated with **NB-360** for 6 weeks had significantly lower concentrations for Aβ38, 40 and 42 than the vehicle group, and were statistically indistinguishable from baseline (Table 4).

**Table 4:** Formic-acid extracted Aβ peptides in forebrain homogenates (pmol/g forebrain)

Aβ species	Mean ± SEM			NB-360 group, change vs baseline	NB-360 group, change vs vehicle
	Baseline	Vehicle	NB-360		
N	13	17	14		
1-38	115.9 ± 38.0	240.3 ± 53.1	77.0 ± 24.9	-33.5 % p = 0.39	-68.0 % p = 0.015
1-40	235.1 ± 37.4	556.1 ± 92.0	197.3 ± 43.4	-16.1 % p = 0.52	-64.5 % p = 0.003
1-42	139.8 ± 25.1	347.6 ± 53.6	106.6 ± 20.9	- 23.7 % p = 0.41	-69.3 % p = 0.0006

**Fig. 5:**
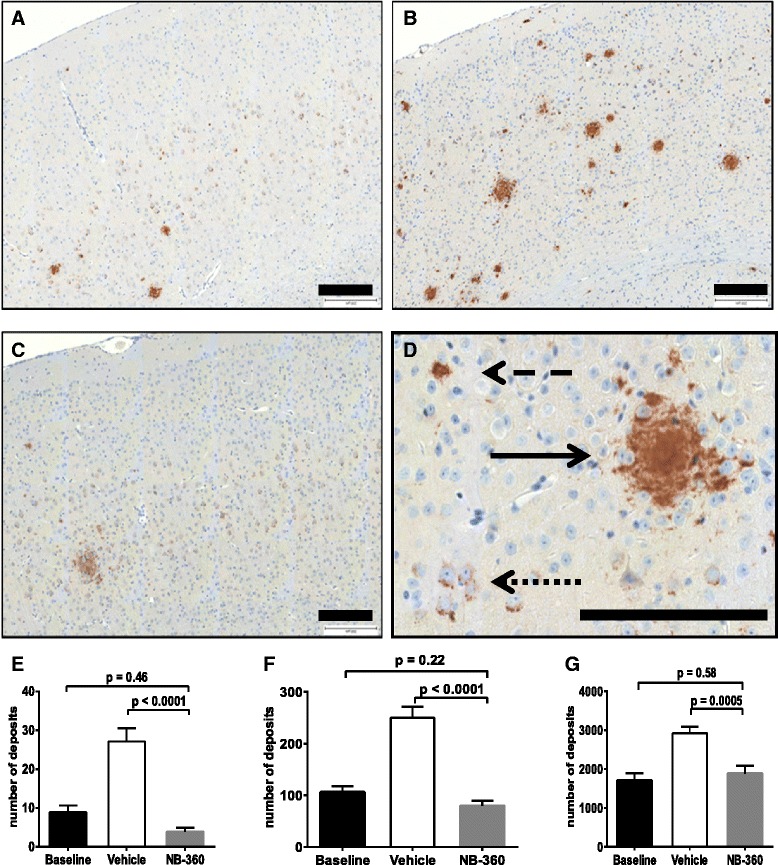
Immunohistochemistry for deposited Aβ of APP51/16 mice; **a**-**c** examples of mouse cerebral cortex. **a** Baseline, **b** vehicle, **c**: NB-360-treated mice. **d** Illustration of the separation of large plaques, small plaques and intracellular granules; continuous arrow > large plaque; dashed arrow > small plaque; dotted arrow > granules. Size bar = 200 μm. **e**-**g** Quantification of cortex area stained positively with NT12 numbers are the sum of deposits in three adjacent sagittal sections, **e** large plaques, **f** small plaques, **g** small deposits

For histological analysis, brain cortical slices were stained with our in house Aβ antiserum NT12. The total area of the cortical region of interest that stained positively for NT12 was significantly higher in the vehicle group (0.754 ± 0.074 %, mean ± SEM), compared to the baseline (0.323 ± 0.031 %, p = 0.0012). There was no statistical difference between the baseline and **NB-360** treatment group (0.209 ± 0.054 %, p = 0.093 vs baseline, p <0.0001 vs vehicle). For a more detailed analysis, Aβ deposits were categorized into large plaques, small plaques and small, mainly intracellular granules, and each size group was analyzed separately and expressed as the number of structures per 3 para-sagittal cortical sections. Irrespective of the deposit size, the vehicle-treated animals showed a substantial increase, compared to baseline, and again the **NB-360** treated animals were indistinguishable from baseline (Fig. 5).

#### Effect of chronic NB-360 treatment on number of activated microglia and astrocytes

**Fig. 6:**
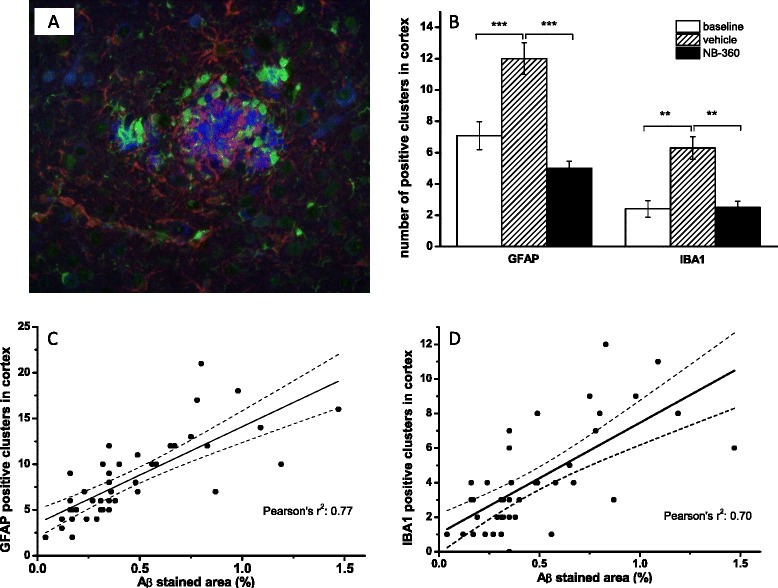
Effect of NB-360 treatment on the number of GFAP-positive astrocytes and Iba-1 positive microglia cell clusters illustrating accumulation of astrocytes and microglia in the vicinity of amyloid plaques. **a** Triple staining of cerebral cortex for aggregated Aβ (blue), GFAP (red) and Iba-1 (green). **b** Quantitative analysis of GFAP+ and Iba-1+ clusters in cortex. Shown are mean ± SEM, **p < 0.01 (Dunnett’s multiple comparisons test) **c**: correlation of total Aβ stained area in cortex with GFAP+ clusters. **d** Correlation of total stained Aβ area in cortex with Iba-1+ clusters. Solid line represents the linear fit, and dashed lines the 95 % confidence interval

We investigated activated microglia cells (stained with anti-Iba1) and astrocytes (stained with anti-GFAP) in APP51/16 cerebral cortex. Triple staining for Aβ deposits and activated astrocytes/microglia cells showed activated cells were present in the vicinity of large, but also small Aβ deposits in the cortex (Fig. 6a). During the 6 weeks treatment period, clusters of neuroinflammation increased substantially in the vehicle compared to the baseline group (Fig. 6b). The number of GFAP positive and Iba-1 positive cell clusters in the cortex was substantially lower in **NB-360** treated APP51/16 mice relative to vehicle-treated mice, reaching values comparable to the baseline group. We also investigated a possible correlation between total plaque load, and the Iba1+ and GFAP+ cell clusters and determined a significant correlation (r^2^ = 0.77 and 0.70 respectively, p < 0.0001). As shown in Fig. 6c and 6d, the number of activated innate immune cells in the brain correlated with the extent of amyloid deposition.

## Discussion

Our optimization of BACE-1 inhibitors in the novel 1,4 -oxazine series resulted in compound **NB-360**, which combines low nanomolar potency on the target with excellent selectivity over the related aspartyl proteases pepsin, cathepsin D and cathepsin E. This combination is an important feature, since treatment of rats with high doses of AMG-8718 caused in retinal toxicity, ascribed to an impairment of the phagolysosomal pathway in the retinal pigmented epithelium by off target inhibition of cathepsin D [12]. Moreover, neuronal lipofuscinosis and degeneration were reported in rats after treatment with LY2811376 [11]. Both compounds inhibit cathepsin D in the low micromolar range (IC_50_: 3.2 and 15.7 μM, respectively). In contrast, the IC_50_ of **NB-360** for cathepsin D is >250 μM and no retina toxicity were observed in the eyes of mice after 8 weeks treatment (data not shown). This suggests that not only the numerical selectivity ratio (IC_50_ CatD/IC_50_ BACE-1), but also the absolute IC_50_ for cathepsin D are important determinant for such off-target effects.

The physicochemical properties of **NB-360** (molecular weight, lipophilicity, and basicity) are in the known range for brain-penetrating compounds. Consistent with its physicochemical and membrane transport properties **NB-360** showed a moderate to high oral bioavailability and an excellent blood-brain barrier penetration as indicated by similar free compound concentrations in brain and in blood. Furthermore, **NB-360** has favorable pharmacokinetic properties, such as good oral bioavailability and low clearance in rodents and in dogs.

The Aβ lowering in brain and CSF of rats was dose- and time-dependent, with the strongest effect at 4 h, while the **NB-360** brain concentration was highest at ≤ 1 h after dose. This is in agreement with the proposed mechanism of action: Inhibition of BACE-1 by **NB-360** reduces generation of new Aβ peptides, but the decline of tissue Aβ depends on its intrinsic clearance, and is independent of **NB-360**. As discussed previously [16], the turnover of Aβ40 in CSF is relatively fast in rodents, compared to higher species. The reported clearance rate of 0.48 h^-1^ for Aβ40 in rat CSF predicts an 85 % reduction after 4 h of complete blockade of the BACE-1 enzyme, in reasonable agreement of the ~90 % reduction observed at the 30 μmol/kg dose. From the dose-dependency a half-maximal efficacious dose (ED_50_) of 1.6 ± 0.25 μmol/kg (0.7 mg/kg) was calculated for **NB-360** showing its high potency *in vivo*. Our design strategy, with emphasis on brain penetration, has led to a compound with the desired level of *in vivo* potency at low peripheral exposure.

A single dose study of **NB-360** at 1.1 μmol/kg (0.5 mg/kg) in dogs confirmed and extended the findings in rats. We observed a good PK/PD correlation, with measurable exposure in blood for 7 days, and the corresponding long-lasting pharmacodynamic effect on CSF Aβ. In agreement with the slower intrinsic Aβ clearance in dog CSF (we found t½ = 3.7 h), it took around 12 h to reach the maximum effect. Based on modeling we could calculate an *in vivo* IC_50_ of **NB-360** in blood (59 nM) for the reduction of Aβ40 in CSF. Overall the dramatically lower compound clearance in dogs versus rats resulted in a considerable prolongation of the pharmacodynamic effect with almost maximal Aβ inhibition still after 48 h as compared to 8 h in the rodents.

Until very recently, LY2811376 and LY2886721 were the only BACE inhibitors with published structure and efficacy data in animals and humans. In PDAPP mice, LY2811376 achieved > 60 % reduction of brain Aβ at doses ≥ 30 mg/kg p.o. At an oral dose of 5 mg/kg in dogs, approximately 70 % reduction of CSF Aβ was achieved. In agreement with these preclinical data, substantial reduction of CSF Aβ in healthy volunteers was seen at oral doses of 30 mg (-20 %) and 90 mg (-54 %) [11]. These results are encouraging, and suggest that efficacy studies with a BACE-1 inhibitor in transgenic mice and dogs have predictive value for the human situation. When compared with LY2811376, **NB-360** scores favorably in terms of dose-related efficacy in preclinical models. Both in transgenic mice and in dogs, a comparable treatment response was achieved with **NB-360** at approximately 10-fold lower oral doses. Since the brain penetration of LY2811376 is comparable to **NB-360**, the better *in vitro* potency of **NB-360** is most likely the reason for the higher efficacy. Very recently, data on the more selective compound LY2886721 were published [10]. The compound dose-dependently inhibited Aβ in preclinical models with similar efficacy as LY2811376 and in human CSF after single and multiple dosing. However, LY2886721 showed an elevation of liver enzymes in a number of AD patients. Liver toxicity is often related to structural features of the parent compound or its metabolites, and may not be a general feature of BACE inhibitors. The structurally different **NB-360** and other new compounds will help to better understand and to resolve this issue.

Previous studies of BACE-1 deletions (BACE-/+ and BACE-/-) in the APP51/16 model have shown a non-linear relationship between BACE-1 enzyme activity and Aβ generation [17]. While heterozygous BACE knock out mice had an about 50 % reduced BACE activity only a moderate (10-30 %) decrease was found for key APP metabolites of the β-site pathway (sAPPβ, C99, and Aβ40). A complete reduction was observed only in the homozygous knock out animals. A study in BACE -/+ PDAPP mice has shown a similar moderate effect of reduced BACE1 activity on neocortical Aβ in young mice (-12 %, [18]). However, at 18 months, total deposited Aβ was reduced by 50 %, in good agreement with the reduction in BACE1 activity. Pharmacological treatment with **NB-360** resulted in 70-75 % reduction in dogs, calculated as an approximate area under the effect curve over a 24 h period from the values at 12 and 36 h. In the dog study BACE-1 inhibition thus must have been far beyond the 50 % of a homozygous knock out for more than 24 h.

Having demonstrated that **NB-360** was a potent inhibitor of Aβ generation in 2 acute animal models of amyloidogenesis, we investigated the effect on Aβ deposition using an APP transgenic mouse model. Internal as well as external data [19] suggest that mice overexpressing the “Swedish” mutation at the BACE cleavage site are less sensitive to BACE inhibitors than models with human wild-type APP, although we achieved substantial Aβ reduction in mice with the “Swedish” mutation (APP23) as well (unpublished data). A recent study with centrally delivered BACE inhibitors in Tg2576 showed significant efficacy in a “Swedish” mouse model [20]. However, as wild-type APP better represents the situation in the majority of AD patients, we chose our wild type APP mouse line, APP51/16, for use in a chronic study. APP51/16 does not carry additional mutation accelerating Aβ deposition, and plaque load is moderate unless the mice are very old. At the age of study commencement (14.5 months) the total amount of forebrain Aβ40 (~230 pmol/g after formic acid extraction) was about 30-fold higher, compared to 4 month old pre-plaque APP51/16 mice (~7 pmol/g, [21]). During the treatment period, total brain Aβ and amyloid plaques increased more than 2-fold in the vehicle group, compared to baseline. However, the group treated with **NB-360** showed no increase of Aβ versus baseline, and neither large nor small plaques were increased. In this regard, no reductions in Aβ or Aβ deposition were found relative to baseline either, it remains an open question as to whether longer treatment may have the potential to remove existing amyloid deposits in this animal model. In addition to the extracellular amyloid deposits, the treatment also reduced the intracellular Aβ staining, which presented as small granules. Although our treatment period was shorter, our data can be compared with the data on RO5508887 [22]. These authors studied the BACE inhibitor in APP transgenic mice carrying the London mutation for 4 months of dosing, and observed a reduction of total deposited Aβ40 by 28 % and of Aβ42 by 45 %, at a dose of 30 mg/kg. Reduction to baseline level was not observed at the higher dose of 90 mg/kg, which may be in part a consequence of the high Aβ42/Aβ40 ratio (~10) which is characteristic for these mice. There is currently no consensus whether or not one should aim for a stabilization of plaque load, or alternatively, for a reduction of the amount of deposited amyloid-β. Furthermore, it is currently unknown to what extent findings in transgenic mice will translate into the human situation. The composition of plaques in human brain may be different from those in APP transgenic mice, and in this respect, mouse data may be of limited value to predict the extent of the treatment response in humans.

To assess the acute effect of **NB-360** we analyzed the secreted metabolites of the β-site pathway sAPPβ and Aβ in CSF from the last dose of **NB-360** in the chronic APP51/16 study. We found a strong reduction in all β-site metabolites at 4 h, which declined by 24 h as observed in pre-plaque mice after a single dose. This indicated that the effect of the inhibitor on Aβ generation remained unchanged over the treatment period of six weeks. In brain a similar kinetic was also observed for sAPPβ and C99. Moreover, a shift of APP processing from the β-site cleavage to cleavage by α-secretase as a consequence of BACE-1 inhibition was demonstrated by the increase in sAPPα and C83. In addition, we found full-length APP was increased by approximately 30 %, suggesting that the competing α-secretase pathway can only partially compensate for this level of BACE-1 inhibition in the APP51/16 mouse model. This pattern of changes in APP metabolites demonstrates that **NB-360** acted as a genuine BACE-1 inhibitor during chronic treatment *in vivo* and confirms the mechanism of action for the Aβ reduction.

Neuroinflammation is another important component of AD pathology, which is postulated to be a response of the brain to amyloid-β deposition [23]. Microgliosis in particular has been described in very early publications as a prominent feature in the AD brain, and there is substantial data supporting the concept that amyloid-induced pro-inflammatory cytokines, reactive oxygen species and other inflammatory mediators play a key role in neurodegeneration, synaptic, and tau pathology in Alzheimer’s disease [24, 25]. We therefore determined the numbers of activated microglia and astrocyte cell clusters in the cerebral cortex of APP51/16 mice as an indicator of brain inflammation. Triple staining of cortical slices showed clusters of both activated microglia cells as well as astrocytes in the vicinity of amyloid plaques already at baseline, the number of which increased in the vehicle group. Treatment with **NB-360** significantly reduced the number of IBA1 positive, activated microglia cells and GFAP positive astrocyte clusters, compared to the vehicle control group, reaching the value of the baseline group. Activated microglial and astrocyte cell clusters showed a clear correlation with amyloid plaques as well as among each other. CD68 staining, specific for phagocytic microglia cells was used in the study of Thakker [21]. The authors found a higher number of CD68 positive cells surrounding the plaques in the treatment group, compared to vehicle. This and our finding regarding microglia are not mutually exclusive and may be related to different treatment times and stages of amyloid formation in both studies. They illustrate our limited understanding of the role and activation status of microglia and/or infiltrating myeloid cells in AD models and in the context of BACE inhibitor treatment. However, both datasets demonstrate that BACE inhibitor treatment also reduces the inflammation present in amyloid plaque bearing brain, and support the notion that neuroinflammation in AD is a response to amyloid-β deposition.

Patches of grey hair appeared on the mice during chronic treatment with **NB-360**. Studies with BACE (-1 and/or -2) knock out mice has implicated both genes in hair pigmentation [26]. Since **NB-360** shows low nanomolar inhibition of BACE-1 and -2, the appearance of grey hairs may be related to the selectivity profile of the compound. The mechanism of this hair discoloration has been investigated further and will be reported in due course. The human relevance of this finding is currently not understood and requires further investigation.

## Conclusion

The data demonstrate that **NB-360** is highly potent *in vitro* and has an attractive combination of drug-like properties, leading to strong direct effects on amyloid pathology *in vivo*. Furthermore, using **NB-360** as an example, we show that, at least in an APP-transgenic mouse model, amyloid-β deposition and neuroinflammation are correlated, and that a BACE-1 inhibitor, which effectively stops further amyloid-β deposition, can also have marked effects on the downstream neuroinflammation. There is a substantial body of literature showing that Aβ plaques are associated with micro-and astrogliosis (reviewed in [27]) and the concept that reducing Aβ may prevent downstream pathology forms the basis of the amyloid hypothesis [28]. Our findings indicate that reduction in amyloid-β via inhibition of BACE-1 may have the desired beneficial downstream effects on Alzheimer’s disease pathology.

## Methods

### *In vitro* protease and Aβ-release assays

Human and mouse BACE-1 as well as human BACE-2 was expressed as recombinant catalytic domain. NB-360 was diluted to final concentrations from 1 nM to 10 μM, incubated with enzyme, and residual activity measured using a FRET substrate with a BACE-cleavable sequence. Cathepsin D and cathepsin E was activated from the corresponding pro-enzymes. Porcine pepsin was the native full-length enzyme. These 3 enzymes were incubated with **NB-360** at concentrations from 1 μM to 250 μM, and residual activity was measured using Mca-GKPILFFRLK(DNP)*D*-R-NH_2_. Percent inhibition was plotted *vs* inhibitor concentration, and the IC_50_ values were calculated using a 4-parameter logistic model. IC_50_ values represent the average of 8 compound batches. Chinese Hamster ovary cells stably transfected with human APP751 (both wild-type and “Swedish” sequence) were used to measure **NB-360** effect on APP metabolism. Cells were incubated with **NB-360** (3 nM-10 μM) for 24 h, and supernatant was analyzed for Aβ40, Aβ42 and sAPPβ using in house or commercial immunoassays.

### Profiling assays

The octanol/water distribution coefficient was derived from the apparent permeability across an artificial liquid membrane [29]. The PAMPA assay was done as described [15]. MDCK cells overexpressing human p-glycoprotein (MDR1-MDCK cells, obtained from Prof. A. Berns, Netherlands Cancer Institute) were grown on HTS Multiwell plates, 1 μm pore size (Becton Dickinson). After formation of a tight monolayer, **NB-360** was applied to either the upper or the lower chamber. After one hour, liquid was removed from the upper and lower chambers and analyzed by HPLC-MS. Apical-to-basolateral and basolateral-to-apical transport rates were calculated. Nonspecific binding of **NB-360** to rat and dog plasma and to rat brain homogenate was determined by rapid equilibrium dialysis as described [30].

### Animals

All animal experiments were conducted with the approval of the Cantonal Veterinary Authority of Basel City, Switzerland. Pharmacokinetic studies used male Sprague Dawley rats, C57BL6 mice and Beagle dogs, obtained from Charles River (France, rodents) and Marshall (Italy, dogs). **NB-360** was dosed intravenously as a solution (NMP/PEG200 based mixtures) whereas for oral dosing, NB-360 was suspended in methylcellulose 0.5 % (w/v) / 99.5 % water/0.1 % Tween 80 and applied via oral gavage. Blood was collected i.v., by sublingual bleeding or at sacrifice whereas a serial blood sampling allowed obtaining individual PK profiles in these species. Blood samples were taken between 5 min (intravenous) and 15 min (oral) up to 24 h (mice), 48 h (rat) and 408 h (dog). Brain exposures were determined in the pharmacological studies by collecting the brain after decapitation at selected time points after dosing.

### Bio analytical methods

Brain samples were homogenized with 2 mM KH_2_PO_4_ buffer (approx. 1/2, w/v). A structurally similar internal standard was added to the blood or brain homogenate samples and NB-360 was extracted by quenching with a fourfold volume of acetonitrile and an aliquot of the supernatant was directly injected into the LC/MS/MS system. **NB-360** was separated from endogenous components using a reverse phase column and a gradient elution with Water/1% formic acid and acetonitrile/1% formic acid. NB-360 was ionized with positive electrospray mode and quantified in MRM mode. The LLOQ ranged between 0.4 and 2 ng/mL in blood and 1.2 ng/g and 6 ng/g in brain.

#### PK data analysis

The absorption and disposition parameters were estimated by a non-compartmental analysis of the mean blood concentration (n = 3 mice) or individual concentration (rats, dogs) versus time profile after oral and intravenous administration.

### Pharmacokinetic/pharmacodynamics studies

Rat studies used male Charles River Sprague-Dawley rats of approx. 300 g body weight. Female transgenic APP51/16 mice (C57BL/6 background strain) were bred in-house. APP51/16 mice express human wild-type APP751 under the control of a Thy-1 expression cassette in brain neurons, and are characterized by mostly cortical, but few vascular amyloid-β deposits. [21, 31]. Mice were used at the pre-plaque stage (<10 mo.) and during the exponential phase of plaque growth (14.5-16 mo.). Dosing was *per os* in a dose volume of 10 ml/kg for mice and 5 ml/kg for rats. NB-360 was dosed either as a hydrochloride salt in water and 2 % cremophore EL (rat dose response and single dose study in pre-plaque APP51 mice) or in all other studies as a free base in a fine suspension in water:methylcellulose:Tween80 (0.5:0.1:99.4). Rodents were deeply anesthetized for CSF sampling from the cisterna magna, accessed through the exposed atlanto-occipital membrane. CSF was harvested into protein lo-bind Eppendorf tubes and frozen on dry ice. Animals were decapitated while anesthetized and blood and brain samples collected. Brains were briefly rinsed in PBS, quickly wet-dissected and samples frozen on a metal plate precooled on dry ice. All samples were stored at -80 ° C until analysis. The dog study was carried out at Charles River Laboratories, Canada. Male dogs were used with n = 4 per group. A cannula with an access port was implanted under anesthesia, to allow serial CSF sampling from the lateral ventricle. Dogs were allowed to recover from the surgery for 2 weeks before the start of the experiment. **NB-360** was applied at 1.5 mg/kg in methylcellulose:Tween 80:water (0.5:1:98.5).

### Brain homogenization and extraction

Frozen rodent forebrains were homogenized in 9 volumes of ice-cold Tris-buffered saline (pH 7.4) containing Complete protease inhibitor cocktail (Roche Diagnostics, Penzberg, Germany) using a Sonifier 450 (Branson) and stored in aliquots at -80 °C. Triton X-100 soluble Aβ was extracted by mixing 50 μl 2 % Triton X-100 with 50 μl homogenate, incubating for 15 min on ice with vortexing, followed by ultracentrifugation at 100000xg for 15 min. The clear supernatant was diluted to a final forebrain dilution of 1:100 and used for analysis. For the extraction of insoluble amyloid peptides, 50 μl forebrain homogenate was mixed with 117 μl of 100 % formic acid and stored on ice for 15 min with vortexing. Samples were neutralized with 950 μl 1 M Tris base, containing Complete protease inhibitor cocktail (Roche, Basle, Switzerland) and stored overnight at room temperature. The supernatant after 15 min centrifugation at 14000 rpm was used for analysis.

### Determination of amyloid-β peptides and APP fragments

Aβ38, 40 and 42 were determined using the electro-chemiluminescence immuno assay kits from Meso Scale Discovery (Rockville, MD, USA) in either singlet or triplex format. Samples and standards were prepared according to the manufactures protocols. The kit based on the antibody 4G8 was used for rat forebrain and CSF samples, and kits based on 6E10 were used for dog CSF or plasma and APP51/16 brain and CSF samples. Soluble APPα and sAPPβ from APP51/16 mice were determined from the 100000xg supernatant and analyzed with Meso Scale Discovery commercial kits. C-terminal fragments C83 and C99 were determined from Western blots. Forebrain homogenates dephosphorylated with lambda protein phosphatase as described [21] and run on a 10 % Tris-bicine gel with 8 M urea. After transfer to Immobilon P membranes (Bio-Rad Bedford MA USA), bands were probed with APP C8 antibody (recognizing the C-terminus of APP) and detected with goat anti-mouse IgG Fab fragment AlexaFluor680 (Invitrogen). The same gel was used to visualize Aβ1-38, 1-40, and 1-42 using the N-terminal antibody beta1 and goat anti rabbit IgG IRdye 800CW (Odyssey). Full-length human APP was detected using an in house immunoassay based on the MSD ECL system as described [17].

### Immunohistochemistry and image analysis

Antibodies and dilutions used were: NT12 (1:1000, Novartis internal rabbit polyclonal against Aβ), 4G8 (1:1000, Covance), anti-Iba1 (1:200; polyclonal rabbit antiserum; WAKO), anti-GFAP (1:5000, rabbit polyclonal DAKO and 1:200 goat polyclonal, Abcam), Secondary detection antibodies: Biotin-labeled goat anti-mouse (DAKO), biotinylated goat anti-rabbit (DAKO), Alexa 594-labeled chicken anti-goat IgG (1:500, Invitrogen); Alexa 488-labeled labeled goat anti-mouse IgG (1:500, Invitrogen), Alexa350-labeled goat anti-rabbit IgG (1:500, Invitrogen).

Automated immunohistochemistry of paraffin sections was performed on 4 μm para-sagittal paraffin sections mounted on SuperFrost + slides and automatically immunostained using the Discovery XT technology (Ventana/Roche Diagnostics). Briefly, sections were de-paraffinized, rehydrated, subjected to antigen retrieval by heating with CC1 cell conditioning buffer, incubated for 60 min at room temperature with primary antibody diluted in antibody diluent, incubated with the respective biotinylated secondary antibody diluted in Ventana antibody dilution, reacted with DABMab kit and counterstained with blueing reagent (Ventana/Roche Diagnostics).

Manual immunofluorescence staining of paraffin sections was performed on 4 μm sagittal paraffin sections. They were de-waxed and subjected to antigen retrieval by microwaving for 10 min at 98 °C in 0.1 M citrate buffer pH 6.0 and rinsed in PBS. Sections were subsequently incubated for 1 h with PBS containing 2 % goat serum, reacted over night at 4 °C with antibody diluted in PBS containing 2 % goat serum, washed 3 times in PBS, incubated for 1 h at room temperature with the respective fluorescent labeled secondary antibodies and mounted with Prolong Gold (Invitrogen).

β-amyloid staining was assessed by digital image analysis. Digital images of slides were captured with a Mirax scanner (Zeiss, 20x magnification) equipped with an Axiocam camera (Zeiss) for fluorescence images, and a Marlin camera (Vision Technologies) for bright-field images. Immunostained plaques and deposits (DAB staining) in 3 para-sagittal mouse brain sections within the cortex were quantified by digital image analysis: For the quantitative plaque evaluation, a proprietary image analysis platform (ASTORIA, Automated Stored Image Analysis) was developed based on MS Visual Studio 2010 and many functions from Matrox MIL V9 libraries (Matrox Inc, Quebec, Canada).

For the beta-amyloid plaque analysis, the following sequence of steps was performed: 1. Slides were scanned with Mirax (20x magnification) 2. Manual outlining of regions of interest defining cortex in brain sections for Aβ plaque assessment 3. Running the in-house developed ImageScope (Aperio Inc., USA) plug-in for creation and export of *.tif image tiles (at 14x mag).

Image batch processing was done as follows: 1.Color deconvolution 2. Detection of nuclei and exclusion of their surrounding from valid plaques 3. Adaptive thresholding technique for segmentation of brown objects 4. Rejection of unspecific granular clusters 5. Feature based object classification into 3 categories: intracellular Aβ (25 - 200 pixels or within nuclear vicinity), small plaques (200 … 1000 pixels and sufficiently detached from nuclei), large plaques (>1000 pixels).

Significance of differences in β-amyloid staining between groups was statistically evaluated by Dunnett’s multiple comparisons test.

### Statistics and calculations

Animals were pseudo-randomly allocated to treatment groups such that body weight difference between groups was minimized. IC50 and EC50 curves were fitted with four-point variable slope dose-response curves. To analyze effects of treatments on groups, data were first tested to determine if they met assumptions for parametric testing (Kolmogorov-Smirnov test (with Lilliefors’ correction) normality test and equal variance tests). Data that met assumptions for parametric analyses were analyzed using 1-way ANOVA followed by the Tukey test for post-hoc comparisons if significant main effects were determined in the ANOVA. Data that did not meet assumptions for parametric analyses (i.e. the time course data in rats) were analyzed using Kruskal-Wallis One Way Analysis of Variance on Ranks followed by Tukey test, as stated above. Correlations were made using Pearson’s correlations. Data were analyzed using Origin (V8.1, MicroCal) or Sigma Plot Version 12.3, (Systat Software Inc.). Two CSF samples were excluded from rat kinetic data analyses because of visible blood contamination (a pre-defined exclusion criterion). Since only n = 4 dogs were used in CSF experiments, data are presented without statistical analysis.

## Abbreviations

AD: Alzheimer’s disease
Aβ: Amyloid β
APP: Amyloid precursor protein
BACE: Beta-site APP cleaving enzyme
CSF: Cerebrospinal fluid
C83 APP: C-terminal fragment, result of α-secretase cleavage
C99 APP: C-terminal fragment, result of BACE-1 cleavage
DAB: Diaminobenzidine
ECL: Electro-chemiluminescence
FRET: Fluorescence resonance energy transfer
GFAP: Glial fibrillary acidic protein
IBA1: Ionized calcium-binding adapter molecule 1
LLOQ: Lower limit of quantification
NMP: N-methyl pyrrolidon
PEG: Polyethyleneglycol
PBS: Phosphate buffered saline
rpm: Rounds per minute
